# Young child with painful edema and purpura: a case report

**DOI:** 10.1186/s13052-021-01001-y

**Published:** 2021-03-10

**Authors:** Sarah Contorno, Giorgio Cozzi, Irene Berti, Egidio Barbi, Andrea Taddio

**Affiliations:** 1 Pediatric Resident, University of Trieste, via del Ponte 1, Piazzale Europa 1, Trieste, Italy; 2 Pediatric Emergency Department, Institute for Maternal and Child Health, IRCCS Burlo Garofolo, Via dell’Istria 65, Trieste, Italy; 3 Pediatric Allergology, Asthma and Dermatology, Institute for Maternal and Child Health, IRCCS Burlo Garofolo, Via dell’Istria 65, Trieste, Italy; 4 Pediatric Clinic, Institute for Maternal and Child Health, IRCCS Burlo Garofolo, Via dell’Istria 65, Trieste, Italy; 5 University of Trieste, Piazzale Europa 1, Trieste, Italy; 6 Pediatric Clinic, Rheumatology and Clinical Immunology, Institute for Maternal and Child Health, IRCCS Burlo Garofolo, University of Trieste Piazzale Europa 1, Via dell’Istria 65, Trieste, Italy

**Keywords:** Pediatrics, Emergency medicine, Dermatology, Vasculitis, Edema, Painful edema, Purpura, Purpuric lesions, Acute hemorrhagic edema (AHE), Vasculitis

## Abstract

**Background:**

We reported the case of a two-old-year boy with a painful acute hemorrhagic edema. This is a self-limited benign condition: usually, affected children are well appearing and this strongly support the diagnosis. In the opposite, in our case, we observed a painful presentation of the edema. Therefore, we demonstrated that rarely, this condition could have also a painful presentation.

**Conclusions:**

This case report helps clinician to know that also acute hemorrhagic edema could have a painful presentation, so we must considered it in the differential diagnosis with sepsis, sickle cell crisis and child abuse. We believe that these findings will be of interest to pediatricians.

## Background

Acute hemorrhagic edema (AHE) is a rare cause of edema and purpuric lesions in children in first years of life [1] ^.^Typically, edema involves upper and lower extremities, sparing the trunk. Purpura has a target-like appearance and involves face, ears and extremities [2]. Usually, AHE is a self-remitting condition with an exclusive cutaneous involvement, but sometimes arthralgia, arthritis and renal involvement may be present [2]. AHE shares the same pathogenetic mechanism of Henoch-Shoenlein Purpura [3]. Both conditions are IgA vasculitis, but both lesions’ pattern and distribution, both age of onset are different [4]. Evidence about treatment is mainly anecdotal, with reports of oral or intravenous steroids administration [5], but there is no clear indication supporting their use.

## Case presentation

**Fig. 1:**
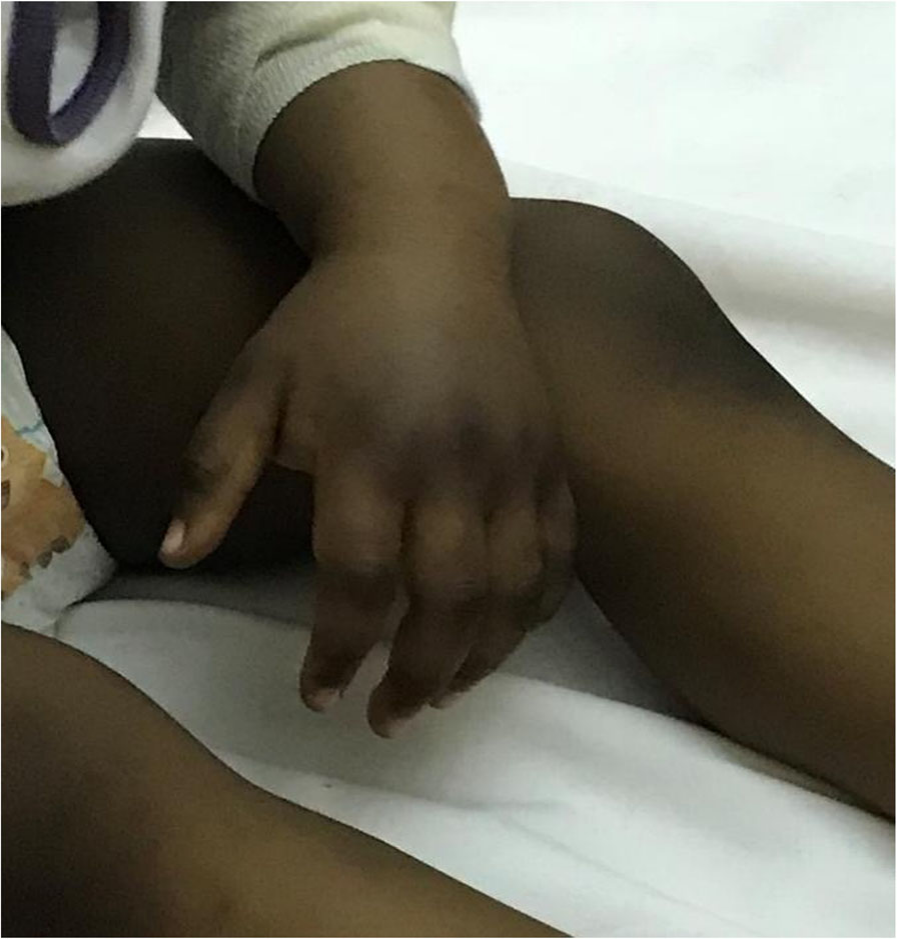
Painful left hand edema

**Fig. 2:**
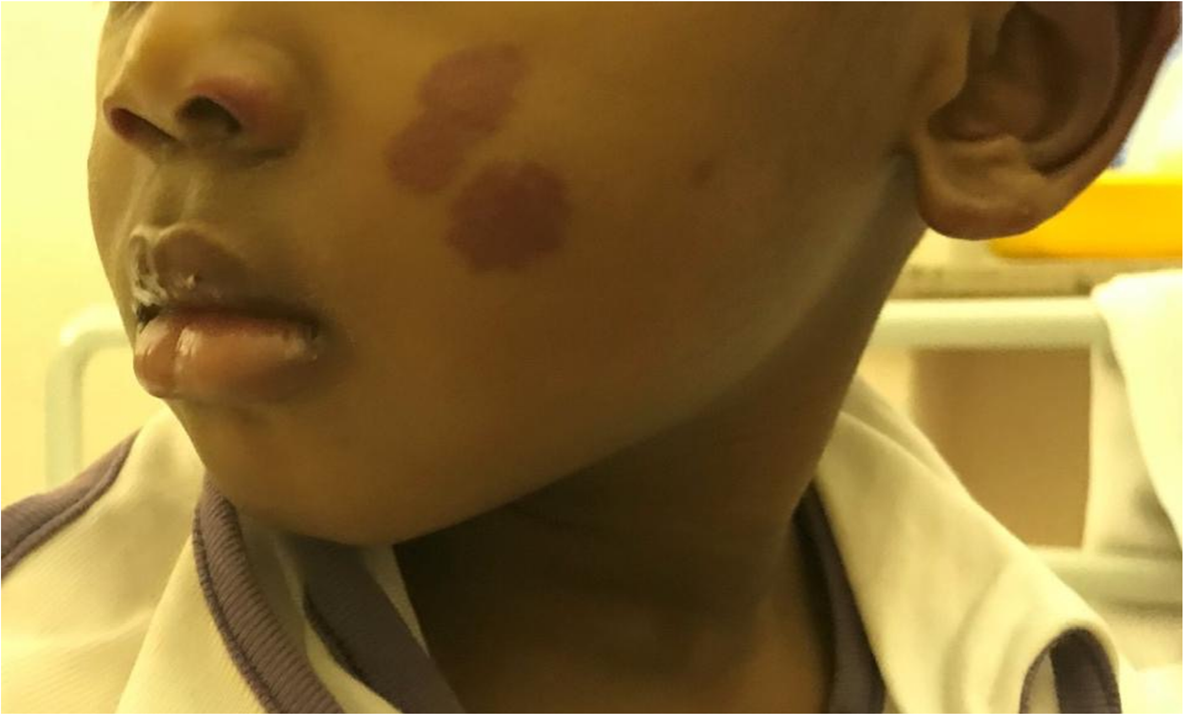
Purpuric lesions on cheek, nostrils and ear

A two-year-old boy presented at the emergency department for an extremely painful bilateral non-pitting edema of both hands (Fig. 1). No fever neither trauma occurred. At examination, the child was well, but he could not use his hands nor walked. Purpuric lesions on the left cheek, nostril and ear were noted (Fig. 2). In the suspicious of an infectious, we performed blood analysis, showing a normal blood cells count and elevated flogosis indexes (CRP 14.2 mg/dL and ESR 110 mm/hr). Urine analysis were negative too. We stated a symptomatic pain treatment with ibuprofen.

**Fig. 3:**
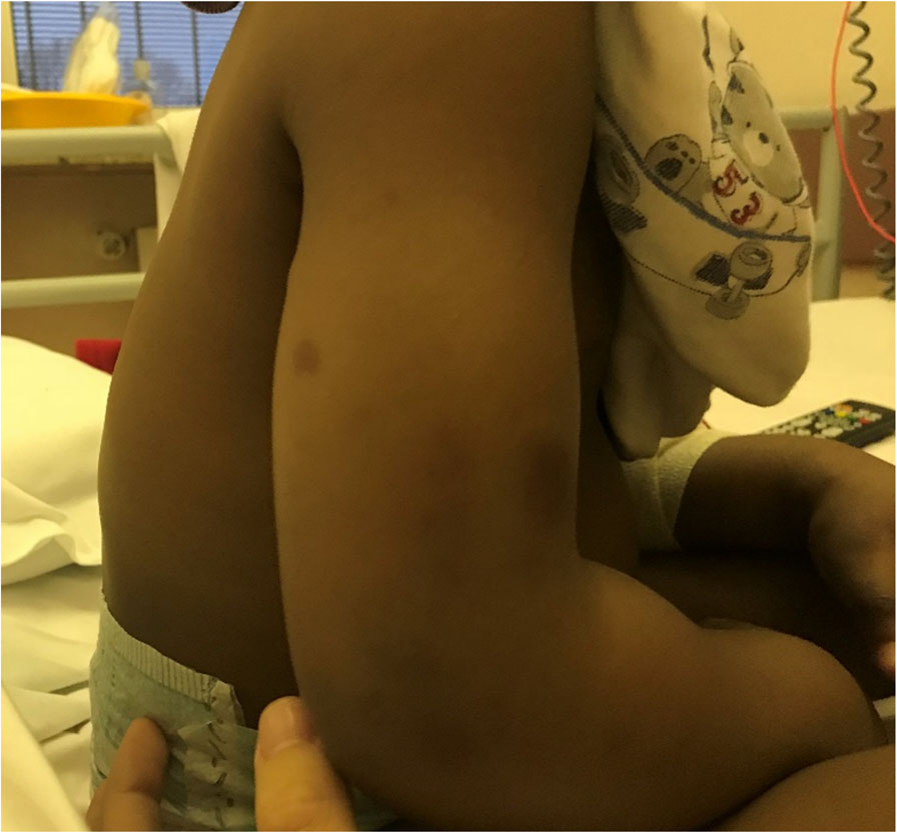
Purpuric lesions and edema on right arm

During observation, both the edema and the purpuric lesions spread to arms, feet and legs (Fig. 3).

We made diagnosis of acute hemorrhagic edema. Our patient received treatment with ibuprofen. Lesions resolved in 72 h.

## Discussion and conclusion

AHE represents a challenge for the pediatrician at the emergency department. Usually, in contrast to striking cutaneous lesions and rapid progression, the overall child general condition is good [6] and characteristically, AHE patients are nontoxic-appearing infants [7]. Usually, in fact, in AHE, affected children are well appearing, and this strongly supports this diagnosis.

In contrast, in our case, the edema was significantly painful and the child very disturbed, so much that he could not use his hands nor walked. Not recognizing the pain as a possible part of this clinical picture, may lead to a misdiagnosis of potentially serious pathologies, which need adequate treatment, such as sepsis, sickle cell crisis, autoimmune thrombocytopenia, coagulopathies or child abuse.

We believe that knowing this unexpected and unusual presentation of the disease could be of interest to pediatricians: in AHE, in contrast to the dramatic cutaneous eruption, clinical conditions are usually optimal, but sometimes they could be badly. Awareness of this may avoid possible misdiagnosis.

## Data Availability

Not applicable.

## Abbreviation

AHE: Acute hemorrhagic edema
